# Ruptured Ovarian Ectopic Pregnancy Managed Laparoscopically With Subsequent Term Delivery After 2 years Follow‐Up: A Case Report and Literature Review

**DOI:** 10.1155/crog/8974058

**Published:** 2026-04-09

**Authors:** Elizabeth Bajomo, Asma Aziz, Mona Sharma

**Affiliations:** ^1^ Early Pregnancy Unit Queens Hospital, Barking, Havering and Redbridge University Hospital NHS Trust, Romford, London, UK, bhrhospitals.nhs.uk

**Keywords:** case report, fertility, haemoperitoneum, laparoscopy, ovarian ectopic pregnancy

## Abstract

**Background:**

An ovarian ectopic pregnancy (OEP) is a very rare form of ectopic pregnancy. Ectopic pregnancies are 2% of pregnancies, and OEP are 0.5%–3% of all ectopic pregnancies, with an even rarer risk of recurrence. Diagnosis is aided via ultrasound and surgical assessment, but confirmation is via histopathology.

**Case:**

We present a case of a woman in her late 20 s (Gravida 2, Para 1—previous normal vaginal delivery at term) with a planned pregnancy who had presented after a private scan to find a right‐sided OEP and free fluid in her abdomen. She had only complained of mild lower abdominal cramping and no pervaginal bleeding. After evaluation, including pelvic ultrasound in our Early Pregnancy Assessment Unit (EPAU) that suspected a right OEP, she had a laparoscopic resection of the right ovarian ectopic tissue, and histopathology confirmed OEP. She recovered well and went home the next day. Serum BHCG returned to normal after repeated testing for 4 weeks, her periods returned to normal after 3 months, and she had a pelvic ultrasound scan 8 months later which showed normal ovaries. Two years later, she conceived naturally and delivered vaginally at term as a low‐risk uncomplicated pregnancy.

**Conclusion:**

An OEP is very rare but can be effectively managed laparoscopically with very good outcomes and fertility preservation. Full‐term delivery outcome observed in this case after 2‐year follow‐up.

## 1. Introduction

The incidence of an ectopic pregnancy is 11 in 1000, and of that 0.5%–3% are ovarian. A primary ovarian ectopic pregnancy (OEP) is a very rare form of ectopic pregnancy. The incidence reported in the literature can range from 1 in 2100 to 1 in 60,000 pregnancies [1–4]. Although rare ovaries can accommodate the expanding pregnancy more readily than the fallopian tubes or other sites of ectopic pregnancy, and so rupture or progression can be diagnosed later. There are, in fact, case reports of OEP diagnosed in the third trimester [4–6]. There is still a potential for serious morbidity and mortality as a consequence of rupture leading to haemoperitoneum and circulatory collapse. Common clinical features are lower abdominal pain, amenorrhoea with or without pervaginal bleeding, but some women can be completely asymptomatic [3].

The risk factors are similar to other risk factors for ectopic pregnancy such as: pelvic inflammatory disease, intrauterine contraceptive device use (more common association with OEP and IUCD in literature), endometriosis, in vitro fertilisation or artificial reproductive techniques (ART) and previous adnexal surgery [2–4]. Higher rates of ectopic pregnancies can be observed in low‐resource regions due to late detection of ectopic pregnancy and late detection and treatment of PID [1].

The pathology involves fertilisation of the ovum in the distal fallopian tube and secondary implantation within the ovary, as a result of failure of extrusion of the follicle. Ultrasound remains the gold standard for diagnosis of ectopic pregnancies, and a transvaginal scan should be performed if a woman is clinically stable. The diagnosis can be aided by Spiegelbery criteria [7] which include: (1) The gestational sac is located in the region of the ovary. (2) The ectopic pregnancy is attached to the uterus by the ovarian ligament. (3) Ovarian tissue in the wall of the gestational sac is proved histologically. (4) The tube on the involved side is intact.

## 2. Treatment

Surgical resection of the involved ovary (preferably wedge resection of the ovary and oophorectomy in the worst case) is shown to be most effective in case reports. This can be either laparoscopic or laparotomy. Laparoscopy is preferred as it results in less intraoperative blood loss, reduced postoperative adhesions, shortened hospitalisation, less requirement for postoperative analgesia and faster recovery [2–4, 6, 8–11]. There are also reports of high rates of successful subsequent pregnancies after surgical treatment [10]. Medical management with methotrexate is less effective as a primary option [2, 3] but effective for cases where there is persistent trophoblastic tissue [3, 4, 9, 12, 13]; therefore, follow‐up HCG levels are important.

This case is rare because OEPs represent <3% of ectopic pregnancies, and successful term delivery following conservative laparoscopic management is infrequently reported.

## 3. Case Presentation

There is written consent from the patient to publish this case report. The patient is a woman in her late 20 s, normal BMI, Gravida 4, Para 1 plus 2. She had suffered one miscarriage, one 3 years ago at 10 weeks and one chemical pregnancy. It was a planned pregnancy and so was very happy to be pregnant and so organised a private scan for reassurance due to her history of miscarriages. She had been suffering mild lower abdominal cramping pain for 3 days but was not of any concern to her. She had no pervaginal bleeding. Her last menstrual period was about 10 weeks ago. Her smears were up‐to‐date with no concerns and no known history of sexually transmitted infections. She was a non‐smoker, not currently on any medication or contraception.

The private scan reported: ‘Uterus anteverted, the endometrium is thickened and echogenic to measurement of 21 mm with no evidence of a gestational sac. The right ovary was bulky and demonstrates two structures. The first is a well‐defined cystic mass measuring 40 mm × 33 mm with thick echogenic walls and central cystic component which contains a 6 mm bean‐shaped echo, no evidence of FH. The second appears to represent a 21 mm × 17 mm corpus luteum. The left ovary was reported as normal. There was free fluid with low‐level echoes noted around the right ovary and in the pouch of Douglas. No adnexal tenderness was reported during the TV exam’. It concluded the appearance is highly likely of a right OEP and the patient was recommended to attend our Early Pregnancy Unit (EPU). She presented promptly to our unit and was reviewed and rescanned for confirmation. It is important to note all of her vital signs were within normal limits and her pain score was zero. Her abdomen was soft and non‐tender on examination. Her serum B‐hCG was 6700 and progesterone 53.6.

**Figure 1:**
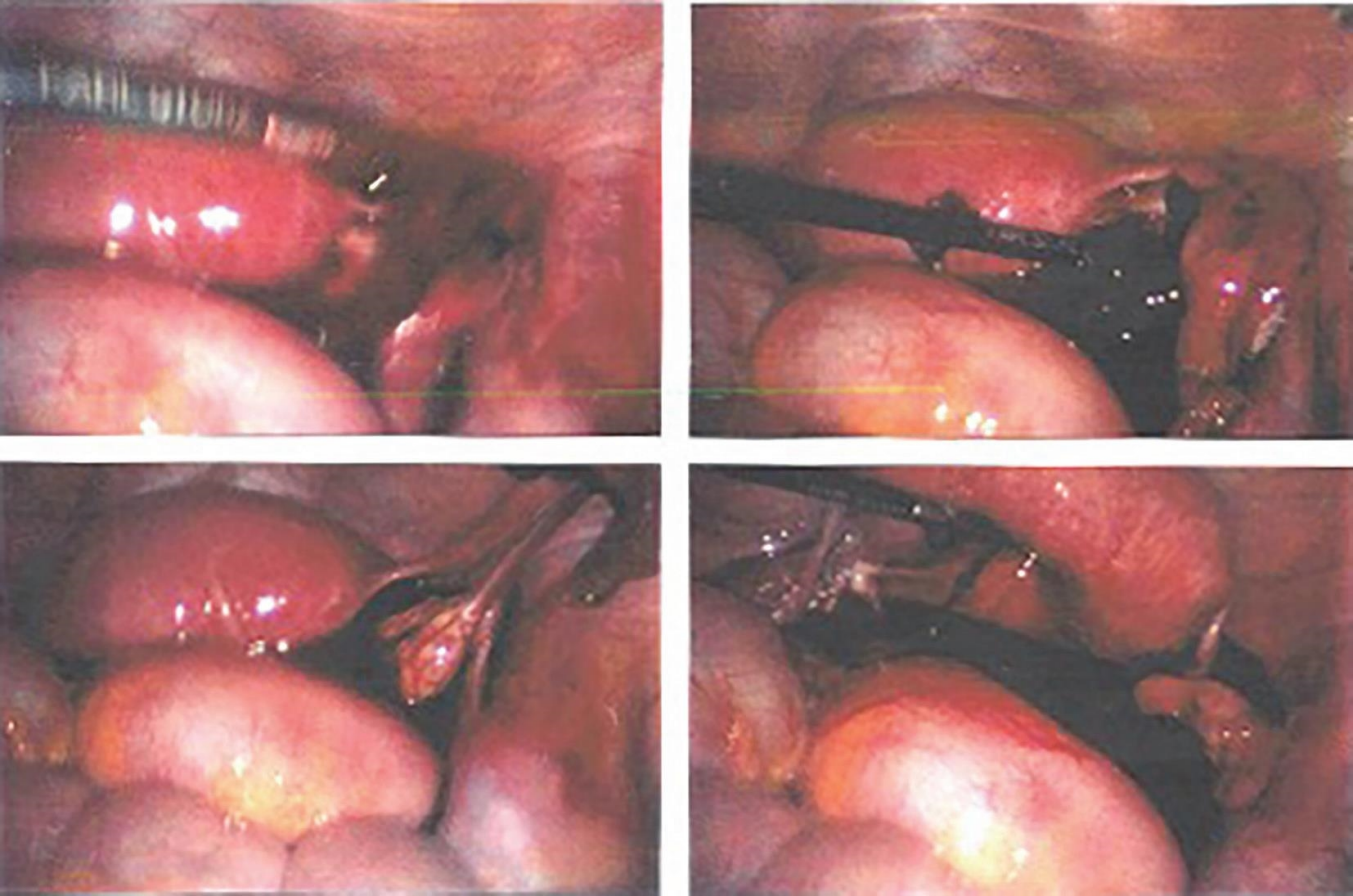
Laparoscopy—right ovarian mass with haemoperitoneum.

**Figure 2:**
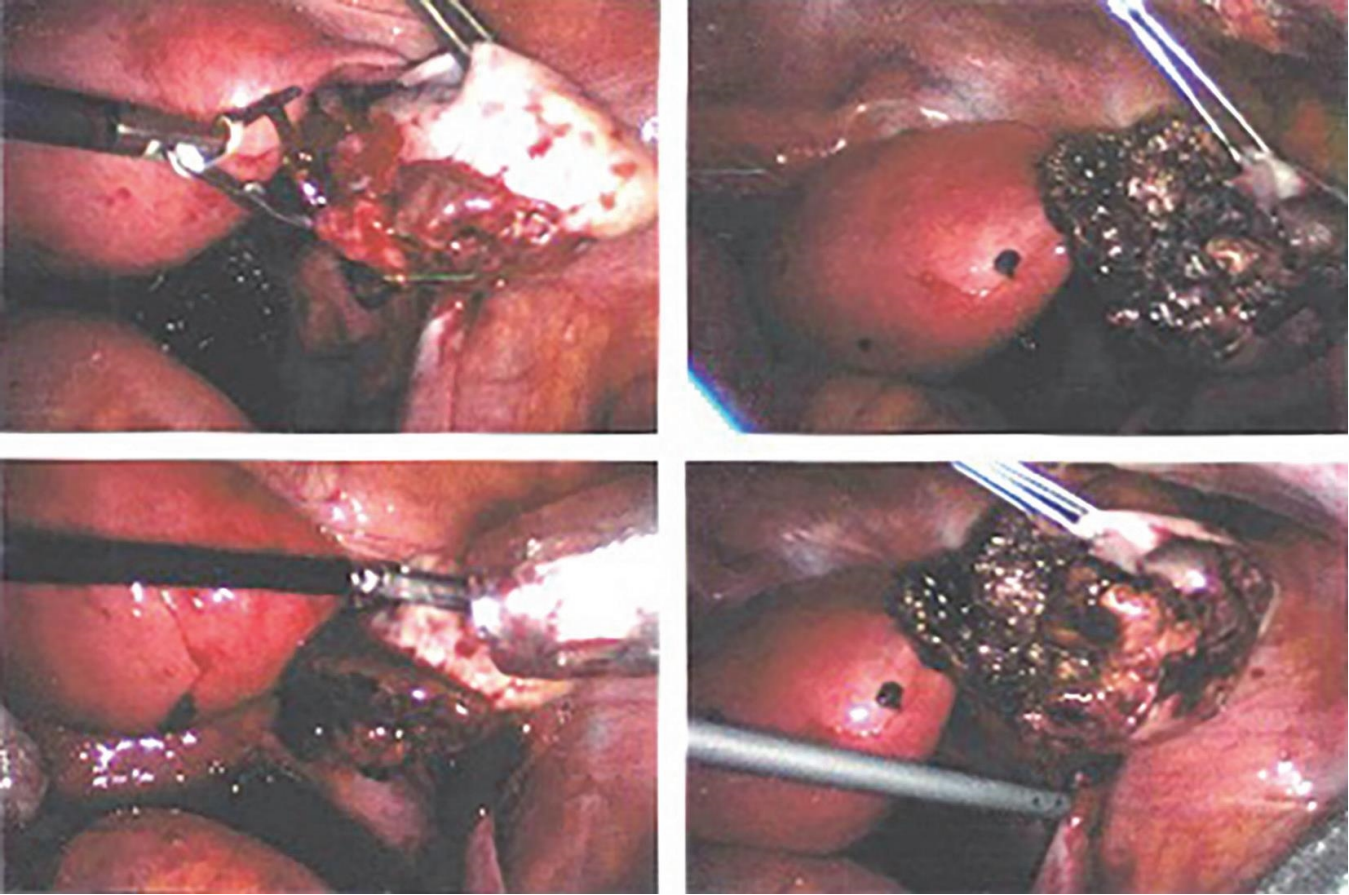
Laparoscopy—right ovarian mass with haemoperitoneum with diathermy applied to the mass.

With the appearance of free fluid and high suspicion of an ectopic pregnancy, the patient was taken to theatre and had a diagnostic laparoscopy. There was about 100cc of blood in the abdomen (Figure 1). The right OEP tissue was resected with diathermy and diathermy applied to any remnant tissue or bleeding points on the ovary. There was conservation of a good amount of the remaining right ovarian tissue. Both tubes and the left ovary were all seen to be normal. Intraoperative blood loss was minimal (Figure 2). The pathology report confirmed trophoblastic tissue within the ovarian parenchyma (Figure 3).

Figure 3(a) Histopathology showing chorionic villi invading ovarian tissue. (b) Chorionic villi invading ovarian tissue. (c) Chorionic villi invading ovarian tissue: lateral view.

**Figure:**
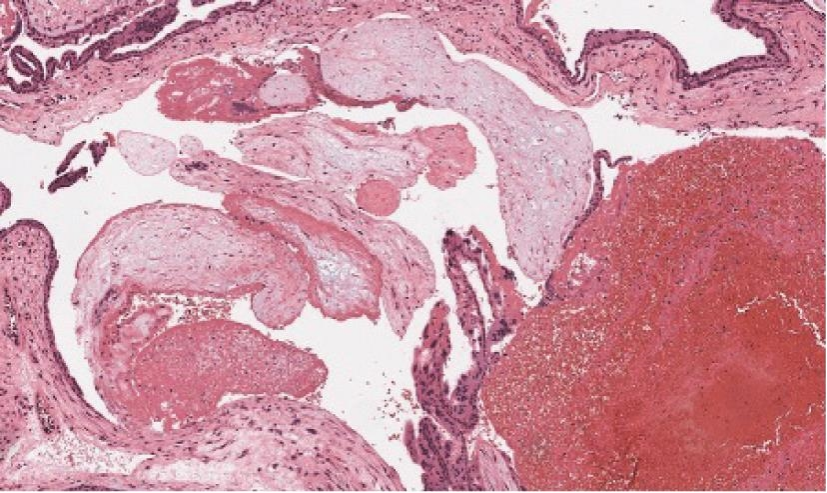
(a)

**Figure:**
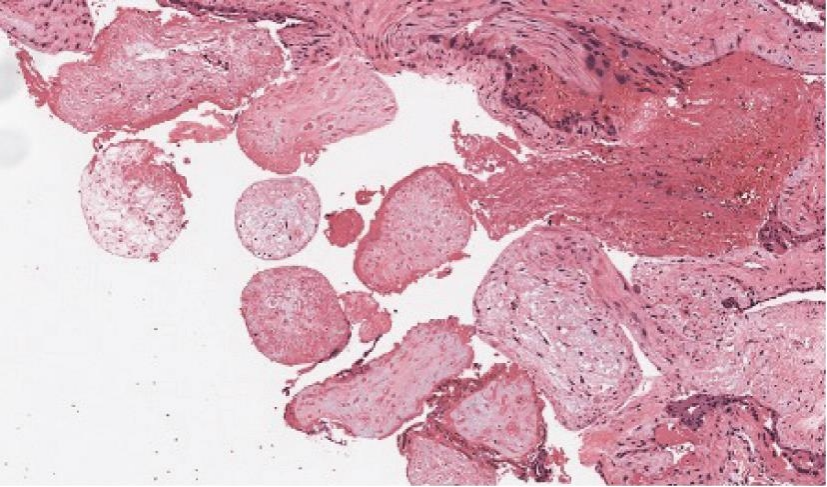
(b)

**Figure:**
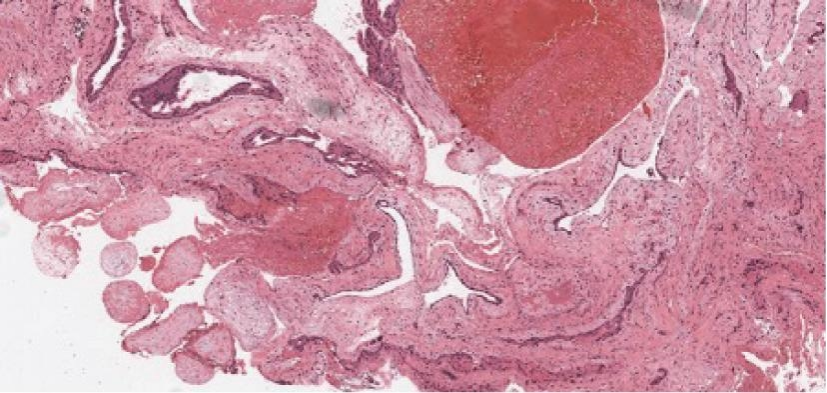
(c)

She recovered well and was discharged the following day with a plan for serial serum B‐hCG monitoring until resolution. The repeat serum B‐hCG on Day 5 and Day 7 were 2180 and 340. The serum B‐hCG returned to <1 after 4 weeks.

Follow‐up with the patient 8 months later, she reported her periods took 2 months to return but returned to her normal cycle and duration thereafter. She was not using contraception as she was hoping to conceive a second child. A repeat pelvic ultrasound scan performed showed normal ovaries and no other pelvic organ issues noted. This demonstrates the effective and successful management of the patient’s case with no complications.

At about 1 year’s follow‐up she conceived naturally and went on to deliver vaginally at full term. Her pregnancy was uncomplicated.

## 4. Discussion

Although rare, OEPs are reported to be increasing in incidence from when it was first reported in 1682, this is thought to be due to the increase in ART, use of IUCD, and better diagnosis with ultrasound and histology [1, 2, 8, 11]. Although ART and IUCD did not apply in this case, it is thought that the association with IUCD is because the presence of IUCD leads to changes in prostaglandin synthesis, which increases tubal motility, leading to implantation in the ovary [14].

OEP can be classified as primary and secondary. Primary OEP results from ovulatory dysfunction, whereas secondary OEP results from secondary implantation in ovarian stromal tissue after tubal abortion or perforation [14].

Here the diagnosis was aided by ultrasound as the patient did not present due to her symptoms. OEP must be differentiated from tubal pregnancies, ruptured corpus luteal cyst and haemorrhagic ovarian cyst [14].

**Figure 4:**
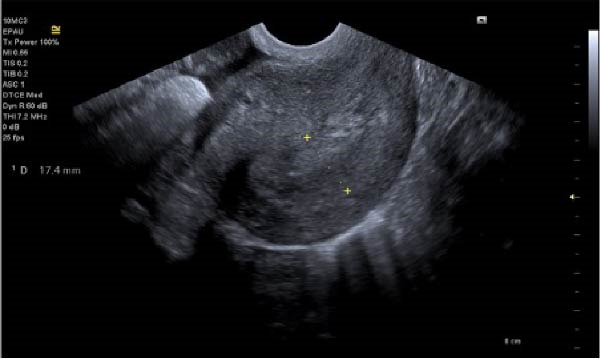
Empty uterus in sagittal view.

**Figure 5:**
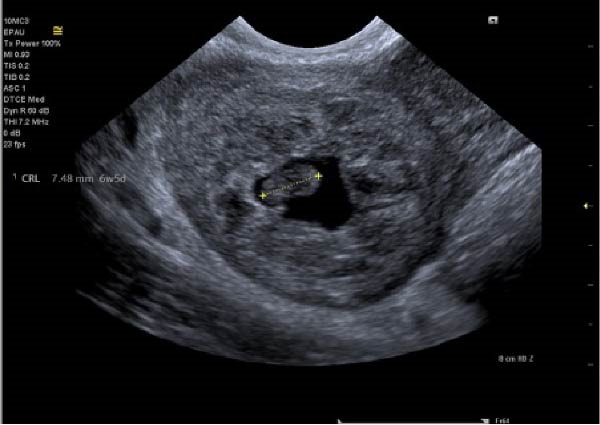
Right ovarian ectopic with fetal pole, no heartbeat.

**Figure 6:**
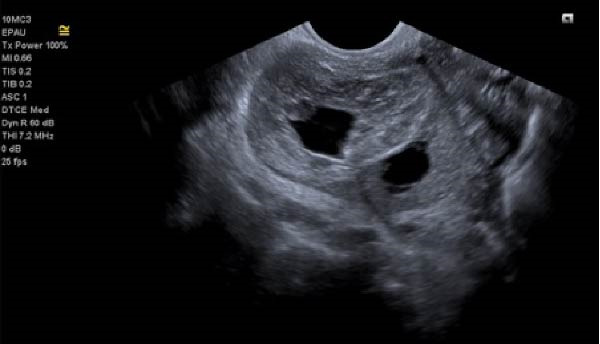
Right ovary showing corpus luteum and ectopic pregnancy.

**Figure 7:**
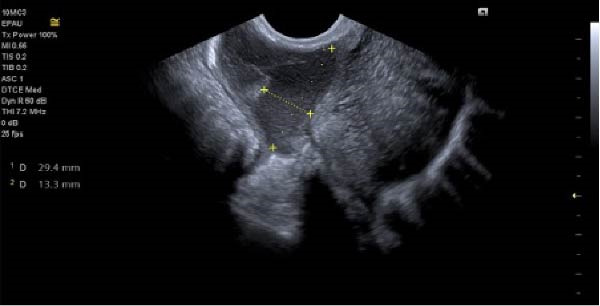
Free fluid in pouch of Douglas.

With ultrasound diagnosis, the features are an empty uterus, a thickened endometrium, free fluid in the pelvis, a complex echogenic adnexal mass or cyst with a wide echogenic outer ring due to developing trophoblastic tissue; this will either be on or within the ovary. Pressure applied via the probe is unable to separate the mass from the ovary. Colour doppler may reveal a hypervascular trophoblastic rim termed ‘ring of fire’ sign, plus or minus the presence of a yolk sac or fetal pole [3]. Although this can be difficult to differentiate from a tubal pregnancy via ultrasound which is why histological confirmation is definitive (Figure 3a, b, c). All of these ultrasound features were seen in this case (Figures 4–7).

All ectopic pregnancies can be managed expectantly, medically [12, 13, 15] or surgically. There is no study yet to prove the effectiveness of management options for OEPs but from published case reports, surgical management is shown to be most effective [3, 4, 8, 16–18]. The surgical techniques used in the management of OEPs are similar to those employed to manage tubal pregnancies. One technique described by Nadarajah et al. [9], involves securing the ovary, followed by sharp or blunt dissection of the ectopic pregnancy off the ovary. Haemostasis is achieved by electrocautery. A similar technique using monopolar or bipolar cautery has also been reported [9]. These techniques generate local ovarian desiccation due to heat production, especially if diathermy use is necessary for haemostasis [16, 17]. This was the method used in this case and was effective in removal of the OEP tissue, preserving ovarian tissues, and ensuring serum B‐hCG returned to normal without the need for further intervention. Her resumption of her menstrual cycle and ultrasound scan 8 months later confirmed resulting normal appearance to ovaries. There are reports of good outcomes following successful treatment of OEP by laparoscopic resection [10], which provided reassurance for the patient as she was planning to conceive a second child. Fernandez et al. [19] in 2013 demonstrated a 2 year in‐utero conception rate of between 64% and 70% after surgical management of ectopic pregnancy. A meta‐analysis by Papageorgiou et al. [20] 2025 shows emerging correlation of higher risk of adverse perinatal outcomes in subsequent pregnancies of women treated for ectopic pregnancy (medically or surgically) such as recurrent ectopic pregnancy, placental abruption, hypertensive disorders of pregnancy and preterm birth. This was not observed in this case.

## 5. Conclusion

There is no randomised trial to establish the best evidence based management option but those used are based on case reports and are shown to have good results, same in this case. In summary, the OEP was well managed resulting in no complications, short hospital stay for the patient. Her follow‐up ultrasound at 8 months showed normal remaining ovarian tissue and she subsequently successfully conceived 1 year later with an uncomplicated term pregnancy and delivery.

## Acknowledgments

The author would like to express special thanks to Dr. P Rajan for the Histology images.

## Funding

No funding was received for this manuscript.

## Disclosure

All authors have read and approved the final version of this manuscript, had full access to all of the data and take complete responsibility for the integrity of the data and the accuracy of the data analysis.

## Ethics Statement

The case complied with the Declaration of Helsinki and local Institutional Ethical guidelines.

## Consent

The patient allowed personal data processing and informed consent was obtained from all individual participants included in this case report.

## Conflicts of Interest

The authors declare no conflicts of interest.

## Data Availability

The authors confirm that the data supporting the findings of this study are available within the article.
